# Supplementary material for: Anti-Inflammatory and Protein Tyrosine Phosphatase 1B Inhibitory Metabolites from the Antarctic Marine-Derived Fungal Strain Penicillium glabrum SF-7123
Source: Mar Drugs. 2020 May 9;18(5):247. doi: 10.3390/md18050247 (PMC7281349; doi:10.3390/md18050247)
Supplement: Supplementary file 1 [file marinedrugs-18-00247-s001.pdf]

Supplementary Material

Anti-inflammatory and protein tyrosine phosphatase 1B inhibitory metabolites from an Antarctic marine-derived fungal strain *Penicillium glabrum* SF-7123

Tran Minh Ha[1,†], Dong-Cheol Kim[1,†], Jae Hak Sohn[2], Joung Han Yim[3,*], and Hyuncheol Oh[1,*]

[1] Institute of Pharmaceutical Research and Development, College of Pharmacy, Wonkwang University, Iksan 54538, Republic of Korea; minhha19@oulook.com (T.M.H.); kimman07@hanmail.net (D.K.)

[2] College of Medical and Life Sciences, Silla University, Busan 46958, Republic of Korea; jhsohn@silla.ac.kr (J.H.S.)

[3] Korea Polar Research Institute, KORDI, 7-50 Songdo-dong, Yeonsu-gu, Incheon 21990, Republic of Korea

* Correspondence: jhyim@kopri.re.kr (J.H.Y.); hoh@wku.ac.kr (H.O.); Tel.: +82-32-760-5540 (J.H.Y.); +82-63-850-6815 (H.O.)

† These authors contributed equally.

List of supplementary data

Content .... Page

Figure S1. HRESI mass spectrum of compound **1** S3

Figure S2. ^{1}H NMR spectrum (400 MHz, CD_3OD) of compound **1** S4

Figure S3. ^{13}C NMR spectrum (100 MHz, CD_3OD) of compound **1** S5

Figure S4. COSY spectrum (400 MHz, CD_3OD) of compound **1** S6

Figure S5. HMBC spectrum (400 MHz, CD_3OD) of compound **1** S7

Figure S6. ^{1}H NMR spectrum (400 MHz, CD_3OD) of compound **2** S8

Figure S7: ^{1}H NMR spectrum (400 MHz, CD_3OD) of compound **3** S9

Figure S8: ^{1}H NMR spectrum (400 MHz, CD_3OD) of compound **4** S10

Figure S9: ^{1}H NMR spectrum (400 MHz, $CDCl_3$) of compound **5** S11

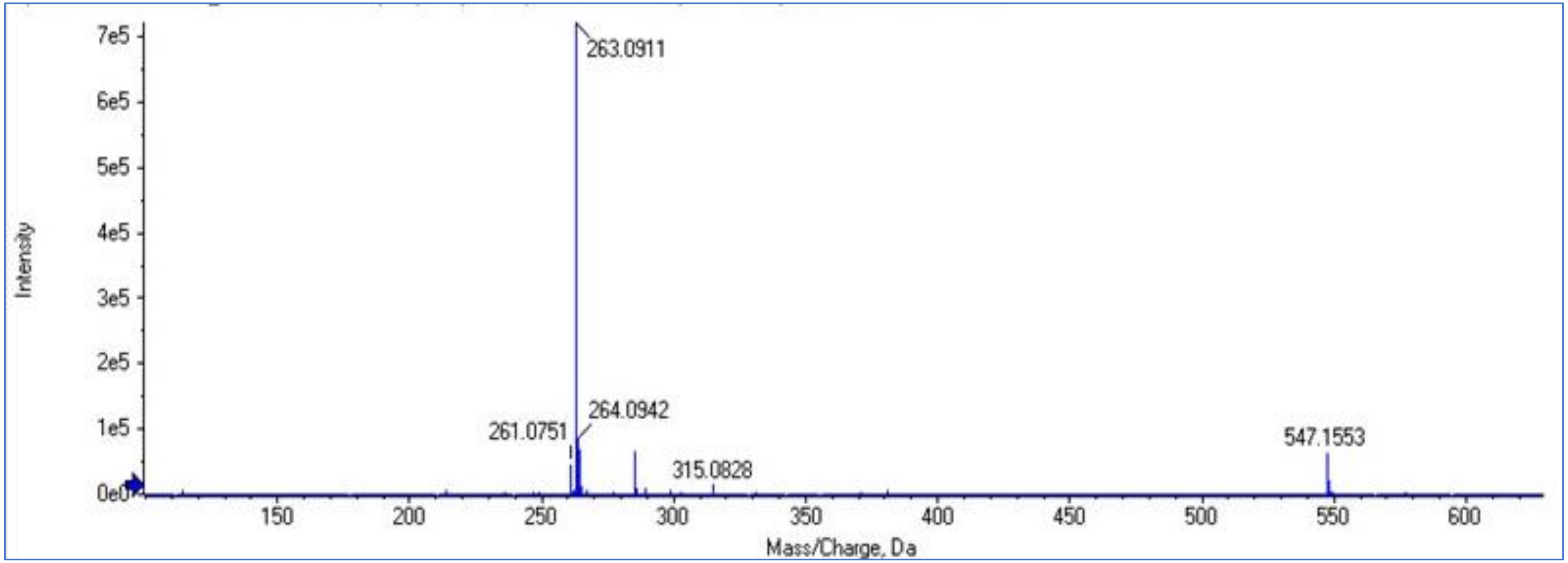

Figure S1. HRESI mass spectrum of compound **1**

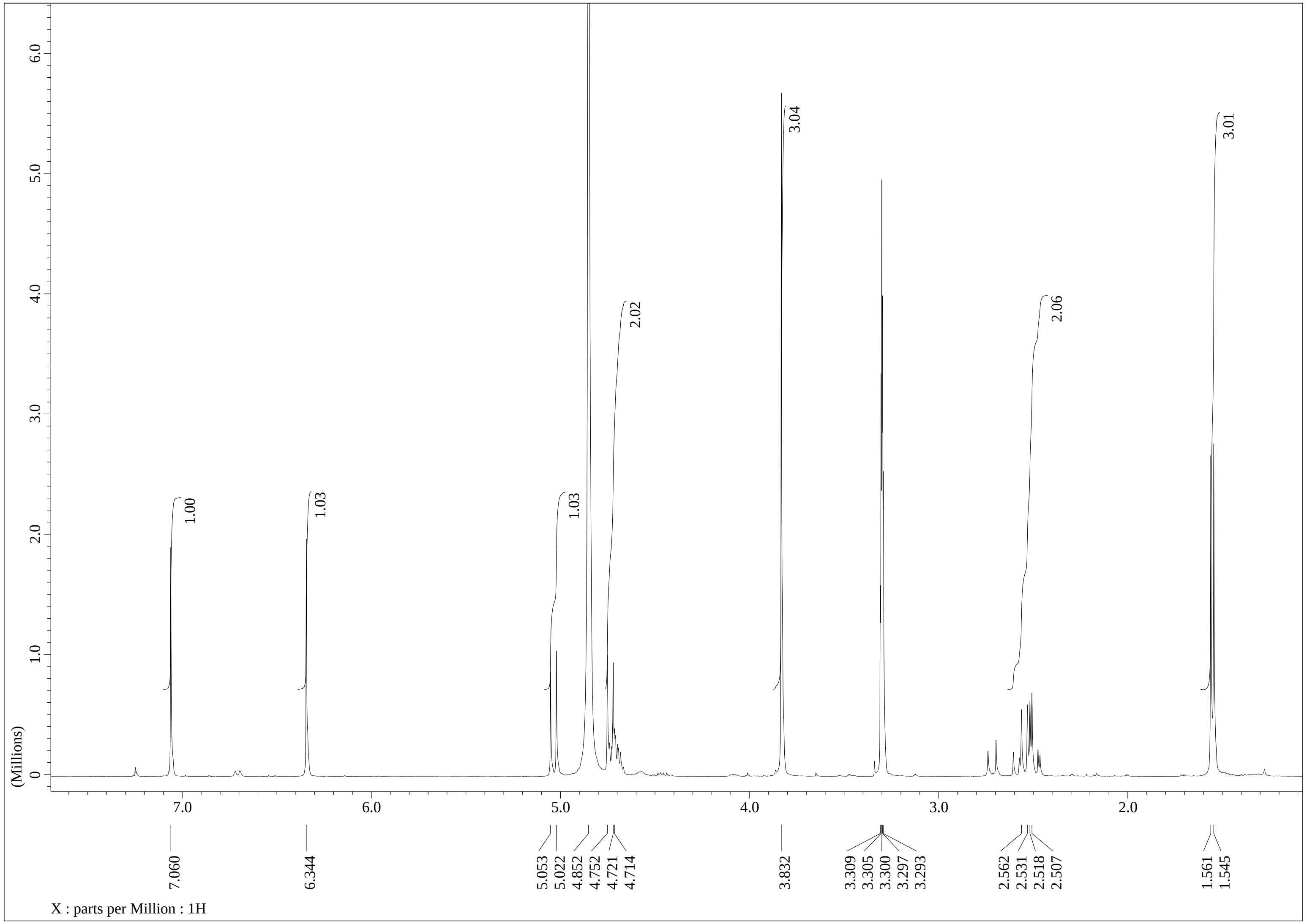

Figure S2. ^{1}H NMR spectrum (400 MHz, CD_3OD) of compound **1**

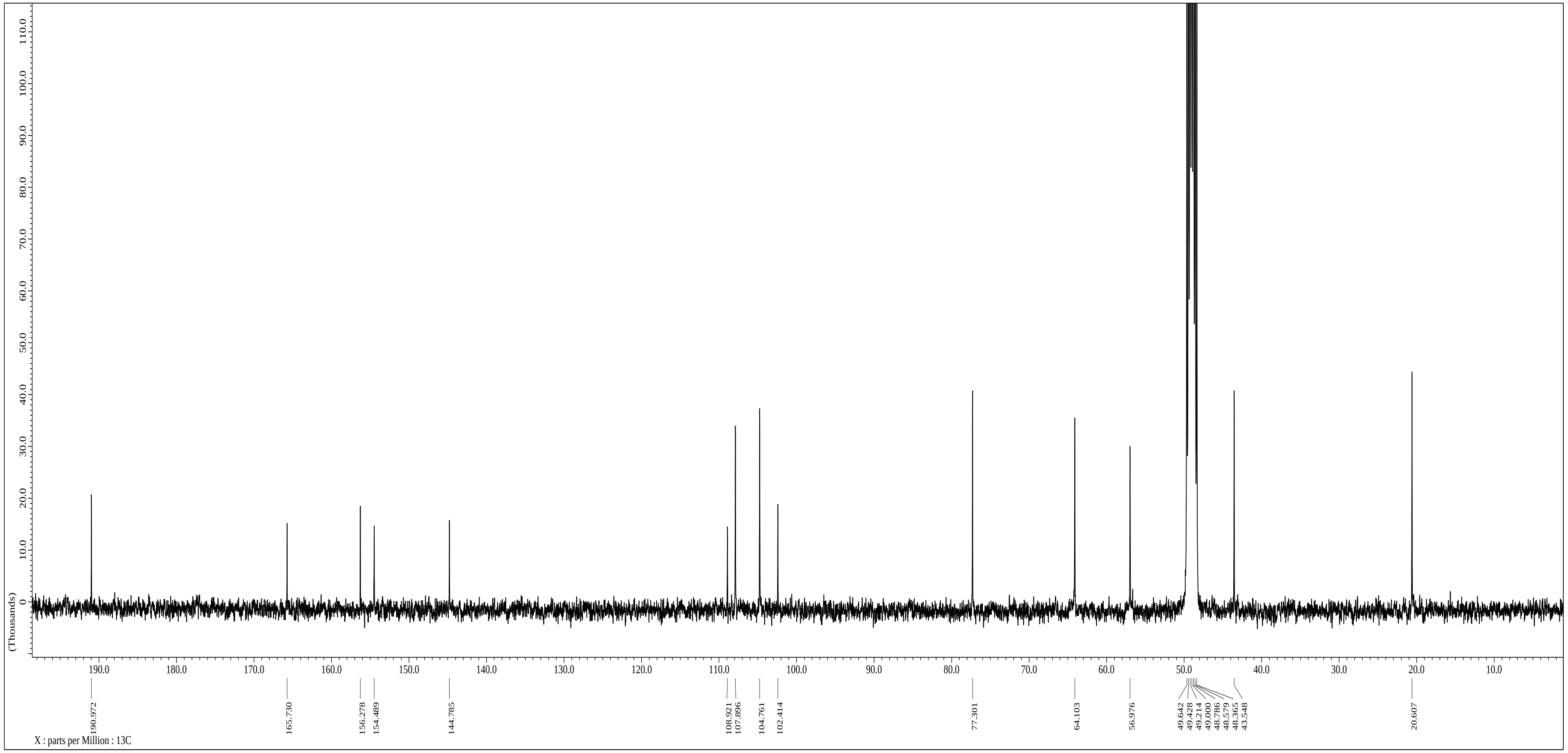

Figure S3. ^{13}C NMR spectrum (100 MHz, CD_3OD) of compound **1**

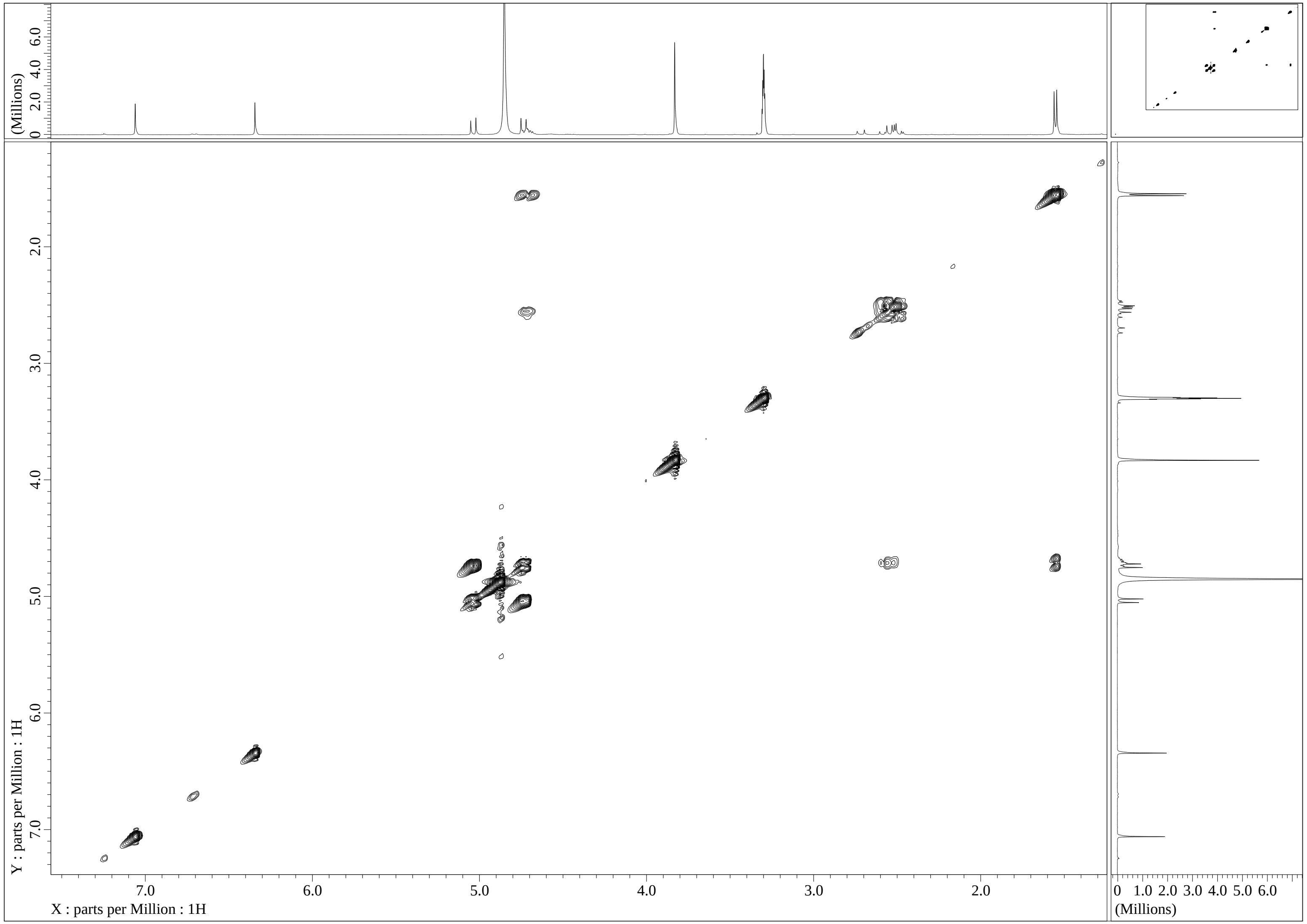

Figure S4. COSY spectrum (400 MHz, CD_3OD) of compound **1**

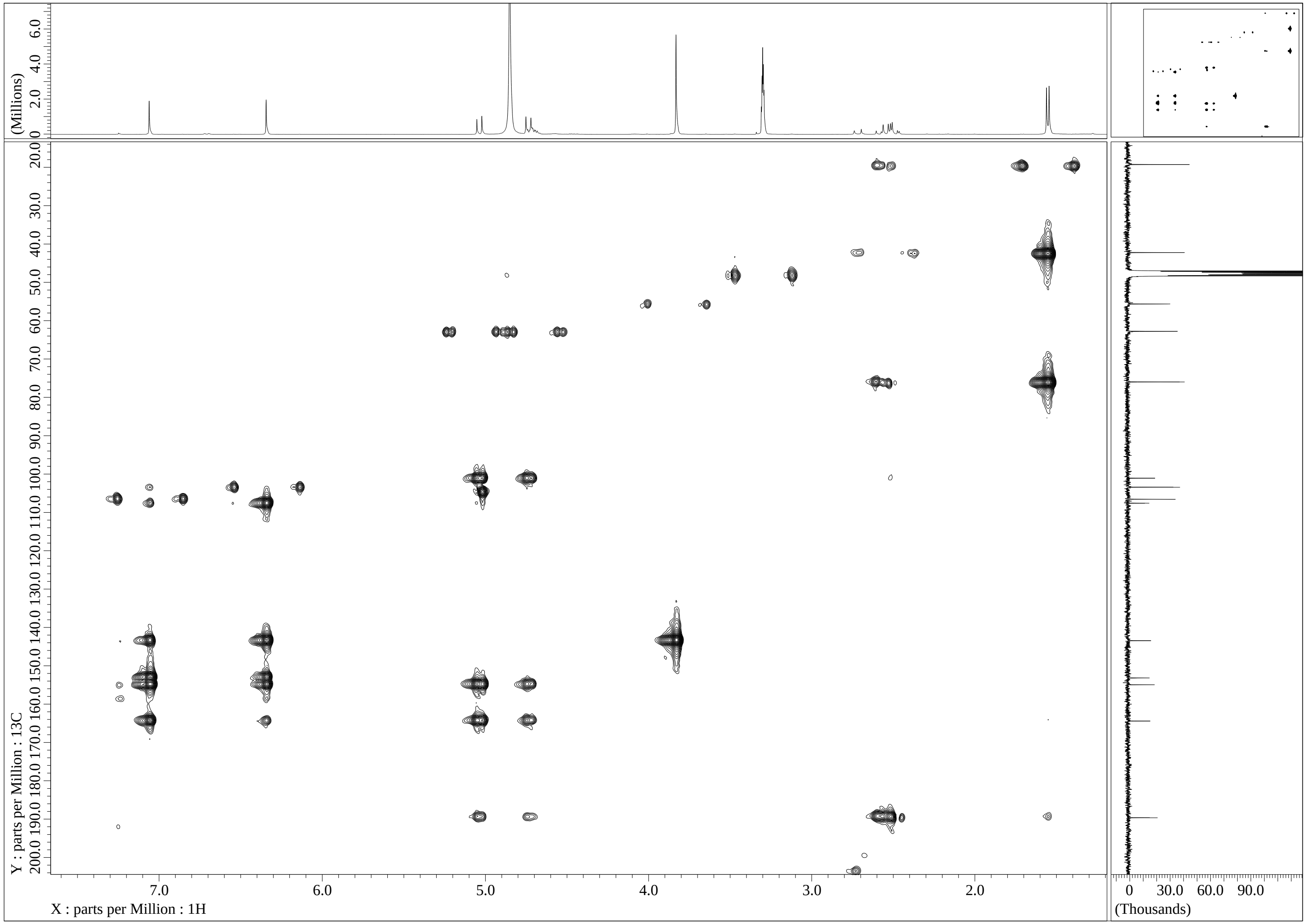

Figure S5. HMBC spectrum (400 MHz, CD_3OD) of compound **1**

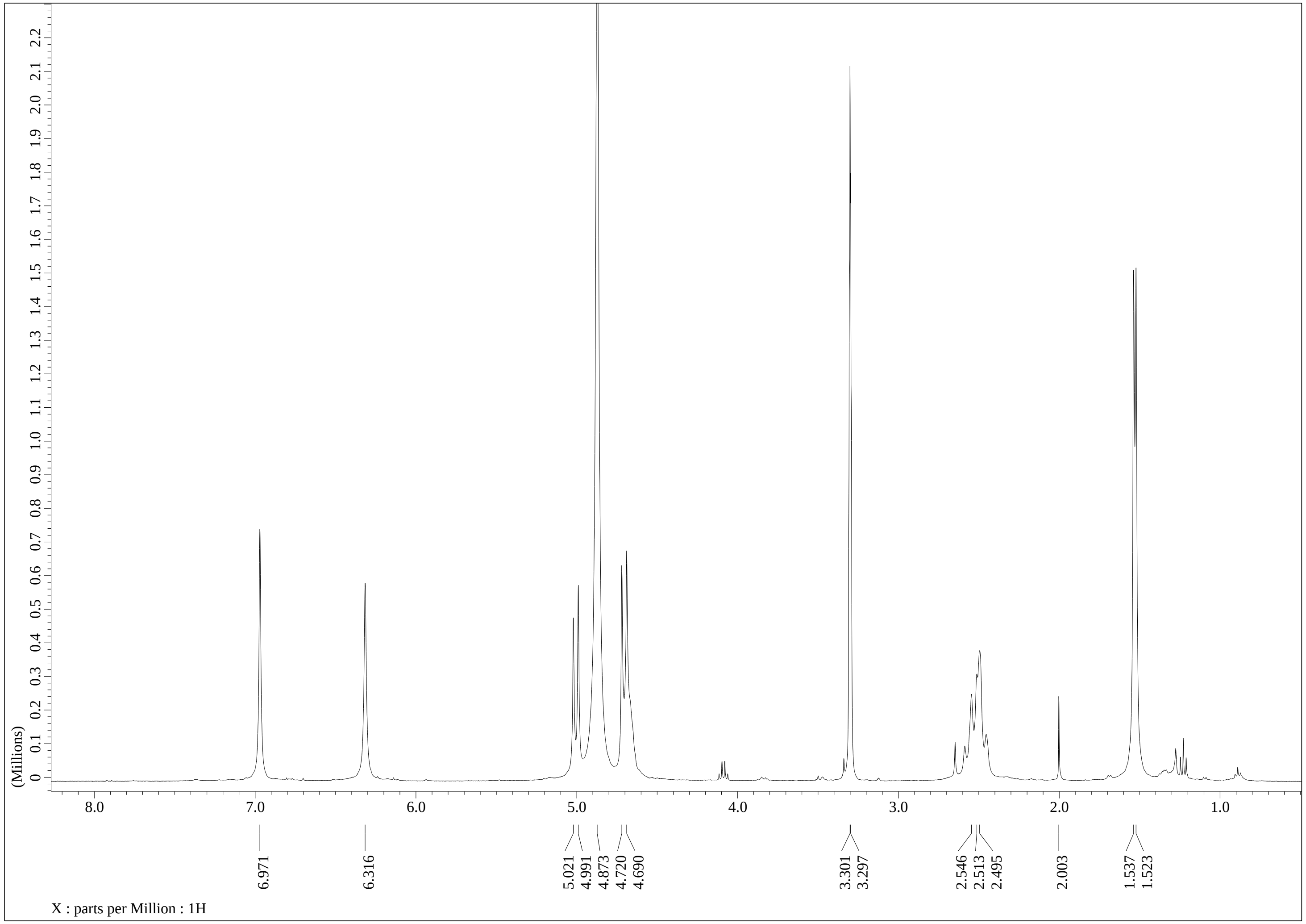

Figure S6. ^{1}H NMR spectrum (400 MHz, CD_3OD) of compound **2**

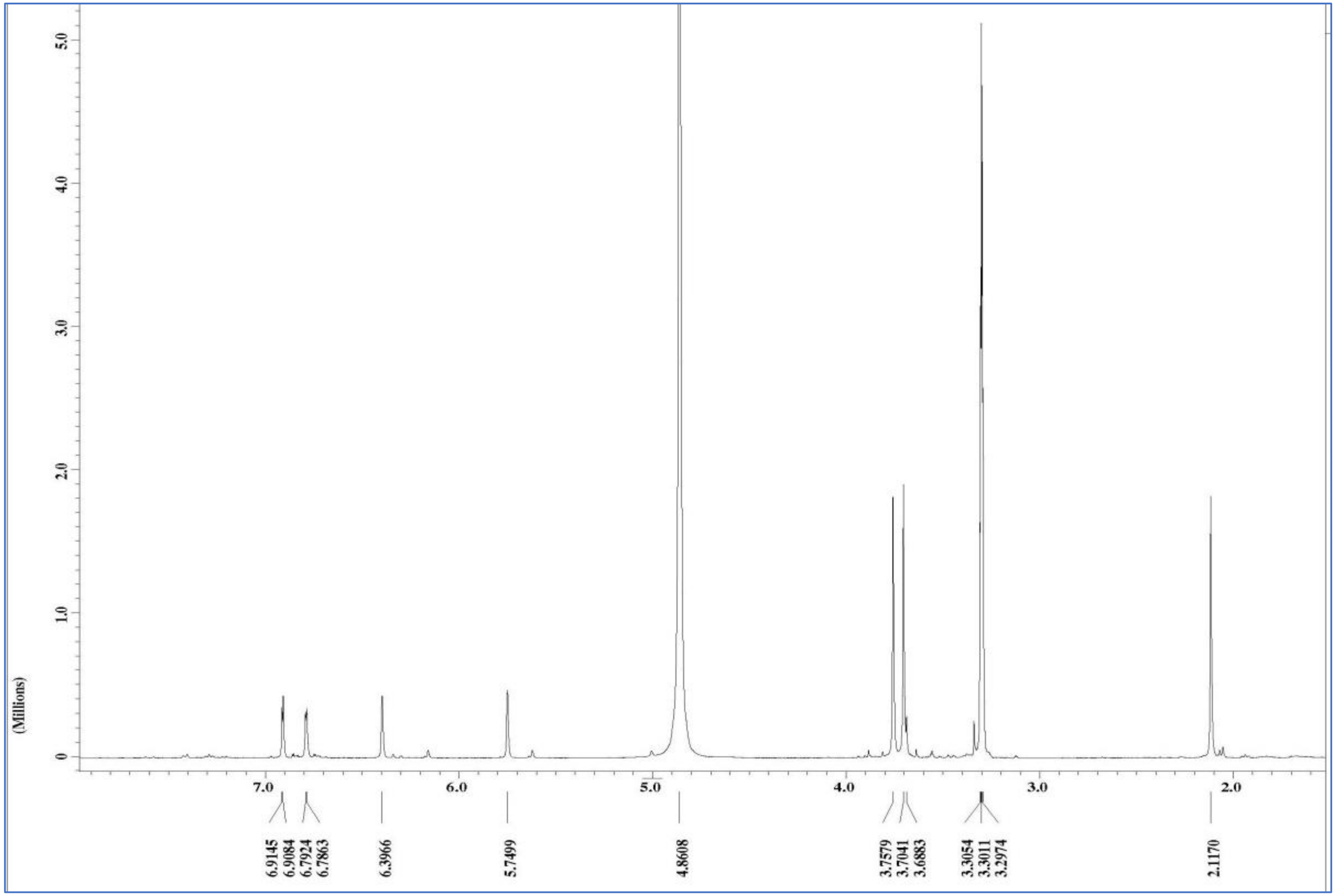

Figure S7. ^{1}H NMR spectrum (400 MHz, CD_3OD) of compound **3**

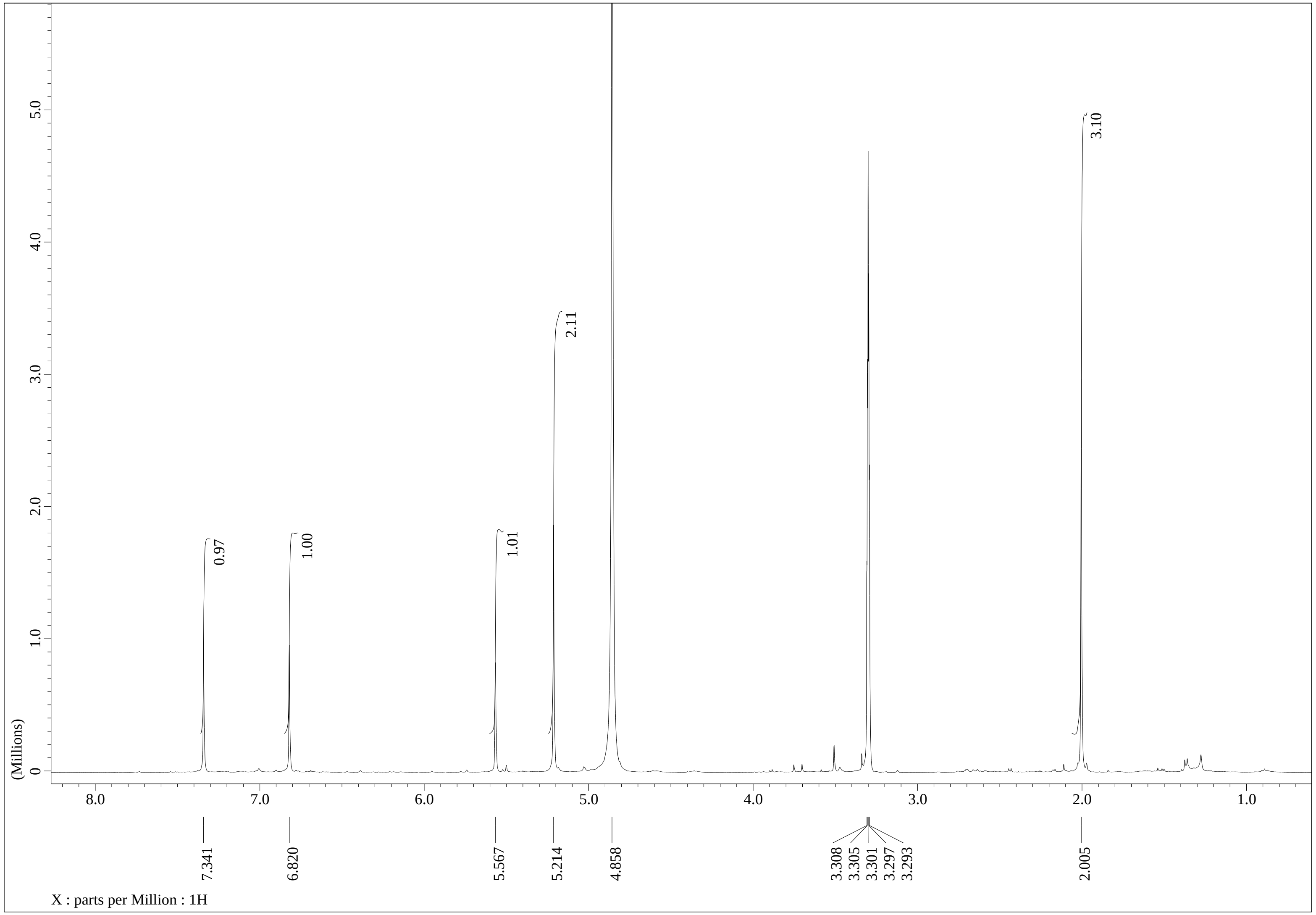

Figure S8. ^{1}H NMR spectrum (400 MHz, CD_3OD) of compound **4**

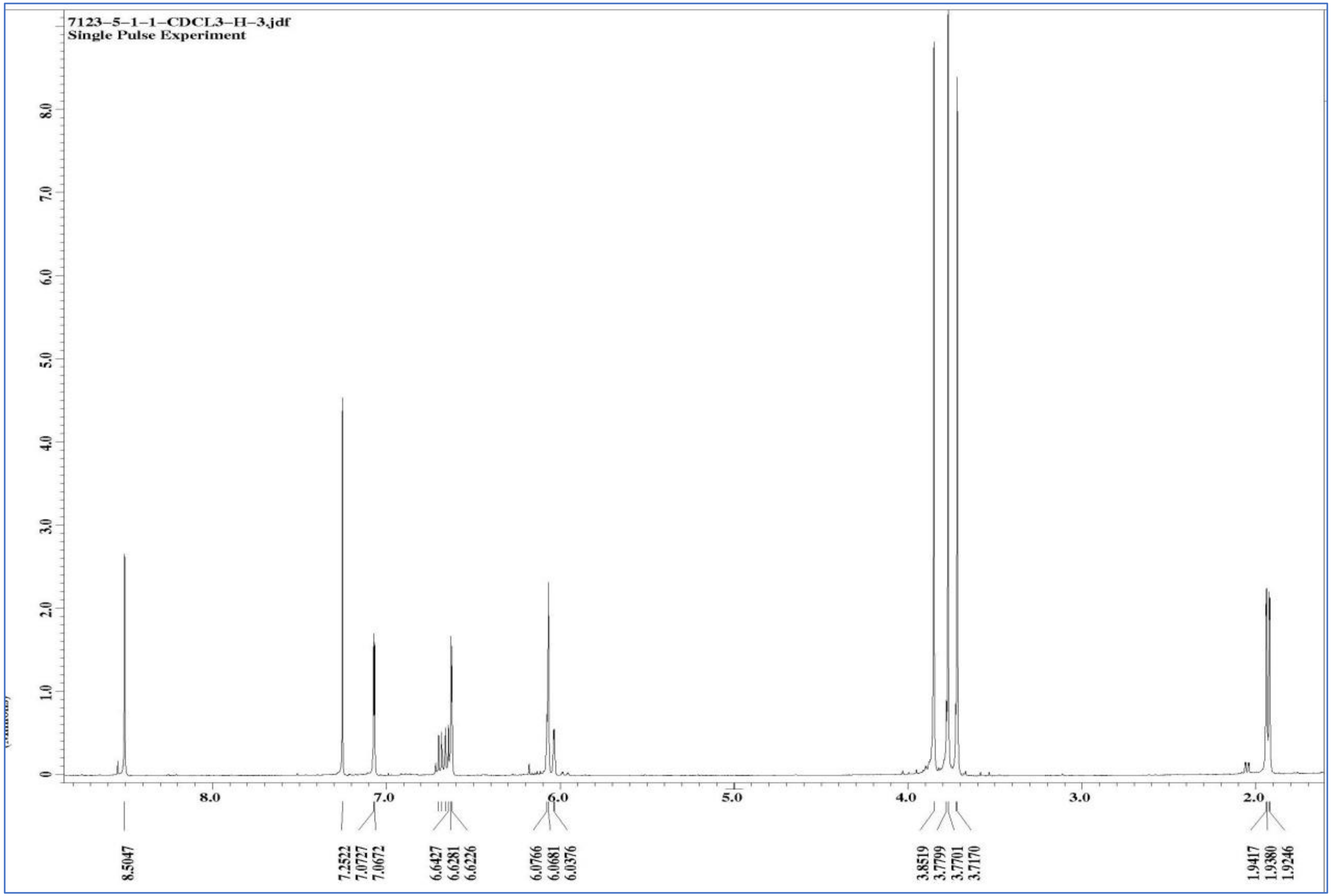

Figure S9. ^{1}H NMR spectrum (400 MHz, $CDCl_3$) of compound **5**
